# Blind UAV Images Deblurring Based on Discriminative Networks

**DOI:** 10.3390/s18092874

**Published:** 2018-08-31

**Authors:** Ruihua Wang, Guorui Ma, Qianqing Qin, Qiang Shi, Juntao Huang

**Affiliations:** 1 State Key Laboratory of Information Engineering in Surveying, Mapping and Remote Sensing, Wuhan University, Wuhan 430079, China; auspicioushua@sina.com (R.W.); xblmars61@163.com (Q.Q.); hjt@whu.edu.cn (J.H.); 2 School of Software Engineering, Huazhong University of Science and Technology, Wuhan 430074, China; shiqiang@hust.edu.cn

**Keywords:** UAV images, image deblurring, image prior, discriminative networks

## Abstract

Unmanned aerial vehicles (UAVs) have become an important technology for acquiring high-resolution remote sensing images. Because most space optical imaging systems of UAVs work in environments affected by vibrations, the optical axis motion and image plane jitter caused by these vibrations easily result in blurring of UAV images. In the paper; we propose an advanced UAV image deblurring method based on a discriminative model comprising a classifier for blurred and sharp UAV images which is embedded into the maximum a posteriori framework as a regularization term that constantly optimizes ill-posed problem of blind image deblurring to obtain sharper UAV images. Compared with other methods, the results show that in image deblurring experiments using both simulated and real UAV images the proposed method delivers sharper images of various ground objects.

## 1. Introduction

Unmanned aerial vehicles (UAVs) represent a quickly evolving technology, gaining attention as remote sensing tools across a variety of scientific fields. In contrast to traditional aircraft or satellite platforms, UAVs have the advantage of lower cost flight missions so they are often employed to produce various photogrammetric and remote sensing products when cost is a factor. Normally, UAVs rarely provide a stable camera platform, which can result in blurred images since UAVs are affected by wind, turbulence, sudden inputs by operators, and by in-flight movements of the aircraft. These blurs impede the visual analysis and interpretation of the data, which can result lower accuracy when using automatic photogrammetric processing algorithms.

Up to now, many deblurring methods concerning remote sensing images have been proposed. Li et al. [1] proposed a remote sensing image deblurring method based on grid computation with distributed processing. Papa et al. [2] used a technique that projects images onto convex sets as a means to establish a priori information in a restoration algorithm for satellite images. Zhao et al. [3] put forward a model containing both total regularization and sparsity regularization terms to deblur and unmix hyperspectral data. Li et al. [4] utilized inadequate exposure motion blur-free image and precise exposure blurred image to obtain blur kernel as a priori knowledge to restore the sharp remote sensing images. Mastriani et al. [5] combined an original technique for noise reduction in the wavelet domain, and a learning approach for Kohonen self-organizing map, to deblur SAR image. Shen et al. [6] used the Huber-Markov prior model to regularize both the image and the blur parameters for the deblurring of remote sensing images. Berisha et al. [7] constructed an optimal Kronecker preconditioner and used spectral data from an isolated star to estimate the multiple point spread functions for joint deblurring and sparse unmixing of hyperspectral image dataset. Liao et al. [8] used PCA transform to separate the information content in a hyperspectral image from the noise and employed a total variation method to jointly denoise and deblur for hyperspectral image. Ma et al. [9] based on compressed-sensing theorem provided a decoding algorithm based on Poisson singular integral and iterative curvelet thresholding to correct the blur problem for remote sensing images. Palsson et al. [10] used a Wiener filter to deblur the images produced by component substitution, multi-resolution analysis, and pansharpening methods. Xie et al. [11] designed an intersect direction iteration algorithm and proposed a total variation restoration model for remote sensing image restoration. Wang et al. [12] estimated the parameters of blurred image based on Bayesian principle to deduce a remote sensing image deblurring algorithm. Tang et al. [13] used displacement vector to build the prior point spread function, then proposed an image deblurring method for remote sensing images based on local temporal compressive photography. Chen et al. [14] constructed a point spread function by high precision motion estimation for remote sensing image deblurring. He et al. [15] used a salient edge selection method based on relative total variation to predict sharp edge information, proposing a deblurring method for remote sensing images. Abrahams et al. [16] chose the optimal Gaussian width to estimate a symmetric Gaussian point-spread function, and then proposed a way mitigating the blurring problem for Defense Meteorological Satellite Program’s nighttime lights images. Cao et al. [17] proposed a deblurring method for remote sensing images based on the relationship between dark channel and convolution. Dong et al. [18] employed the standard Richarson-Lucy algorithm with a piece wise local regularization term and combined it with residual deconvolution method to restore remote sensing images. Jidesh et al. [19] provided a levelset-driven anisotropic diffusion model for deblurring of SAR image, formulated using a non-local regularization framework.

The traditional image deblurring can be divided into blind and non-blind deconvolution. Non-blind image deconvolution can be carried out in various ways, but these methods all require additional knowledge. Yu et al. [20] detailed a deblurring method for remote sensing images that built a multi-scale pyramid image based on local region selection. Xu et al. [21] used the orbit and camera parameters to estimate the extent of lunar image motion blur and image motion value from the blurred lunar image based on small crater detection scheme, adopting the regularization method to deblur the lunar remote sensing images. Some researchers gained additional information through a variety of methods including fluttering shutters [22], color channel dependent exposure times [23] and video cameras [24].

Blind image deconvolution uses only the blurred image and no additional information, and remaining complete the task of deriving a sharp image. In recent years, progress has been made in blind image deconvolution [25,26,27,28,29,30,31]. Cho et al. [32] utilized a shock filter with bilateral filter together to predict sharp edges, and then selected the salient edges for kernel estimation. Xu et al. [33] proposed a effective mask computation algorithm to adaptively select useful edges for kernel estimation and the iterative support detection method was introduced to refine blur kernel. Shan et al. [34] proposed a piece-wise continuous function to fit the natural image gradient distribution. Many image priors have also been introduced that favor clean images clean over blurred images. Krishnan et al. [35] used Hyper-Laplacian distribution to approximate the natural image distribution. Wang et al. [36] employed image edge information as prior for blind motion deblurring. Dong et al. [37] constructed prior knowledge from experts for blind image deconvolution. Michaeli et al. [38] exploited internal patch recurrence to recover the underlying blur kernel. Pan et al. [39] utilized a dark channel prior to blind image deblurring. Xu et al. [40] proposed a L0-regularized prior for image deblurring.

Currently, convolutional neural networks (CNN) have emerged as a promising method to automatically learn deeper feature representations from images. They successfully obtain remarkable results in image processing [41,42,43,44,45,46,47,48]. Generative Adversarial Networks (GAN) have been proposed to synthesize realistic images by effectively learning the distribution of training images [49]. In order to distinguish between real images and generated images, the discriminative ability of GAN is constantly enhanced through training. GAN [50,51,52,53,54], whose optimization methods are constantly being improved, have been also applied widely in image generation and classification. 

In the proposed method, we employ the powerful distinguishing ability of GAN to establish networks that effectively distinguish between blurred and sharp UAV images. In our approach, we take the trained networks as a regularization term in the maximum a posteriori framework, which as a prior information, is used to continuously optimize the blind deblurring of UAV images to obtain better deblurring results. In order to make the discriminative model provide more useful prior information for UAV image deblurring during training, we input blurred UAV images of different intensities into the model so that the trained model will become more robust when faced with different blurred UAV images. The results from deblurring experiments with real blurred UAV images show that the proposed method yields sharper deblurring results than the other methods tested.

## 2. Background

The blur process of UAV images can be generally modeled as Equation (1):

(1) B=L⊗f+n

where, *B* is a blur UAV image, *L* is a latent image, *f* is blur kernel and *n* is the image noise. Blind UAV image deblurring is an ill-posed problem because the number of unknowns exceeds the number of observed data. An observed blurred image provides only limited constraint on the solution, so there are many possibilities for obtaining a sharp image from observed blurred image, which requires regularization to solve. In order to solve this problem, prior knowledge for both UAV images and blur kernels is essential.

Many methods estimate the latent image *L* and the blur kernel *f* from the blur image *L* based on Equation (1), which can be expressed as Equation (2) [39,40]:

(2) $$\underset{L,f}{\min}∥L⊗f-B{∥}_{2}^{2}+\gamma ∥f{∥}_{2}^{2}+\mu ∥\nabla L{∥}_{0}^{}+\lambda P(L)$$

where, the first term indicates the convolution output of the deblurred image, furthermore, and the blur kernel should be similar to the observation; the second term is used to regularize the solution of the blur kernel; the third term is *L*_0_ gradient prior as a regularization term [40]; the fourth term is used to measure sparsity of the priors of latent image. The critical element to this framework is the latent image prior, and clearer UAV image is more helpful for the minimizing Equation (2) to solve the ill-posed problem.

## 3. Proposed Method

### 3.1. The Process of Obtaining Image Prior

In order to better solve the blur problem in UAV image, we present a new method to learn an image prior based on discriminative networks. GAN [49] is a novel deep learning method based on CNN, which adopts a min-max adversarial game theoretic optimization framework and has powerful ability of image generation by continuously updating discriminative ability of true image and false image, as shown in Equation (3):

(3) $$\underset{}{\underset{D}{\min}\;\underset{G}{\max}}\;E_{x∼p_{r}}[\log(1-D(x))]+E_{x∼p_{g}}[\log G(x)]$$

where, $p_{r}$ is the sample distribution of the actual image, $p_{g}$ is the sample distribution of the generated image.

Through continuous training, the probability distribution of the image generated by the generative model is indistinguishable from the probability distribution of the true image, which makes it possible to generate clearer UAV images from blurred ones. Meanwhile, the continuous training also leads to continuous enhancement of the classification ability of the discriminative model. Because the image prior has higher sensibility for blurred images and lower sensibility for clear images, we can use the effective discriminative ability of GAN as prior image, which acts in a trained classifier as a regularization term of the latent image for UAV image deblurring.

#### 3.1.1. The Structure of Discriminative Networks

**Figure 1:**
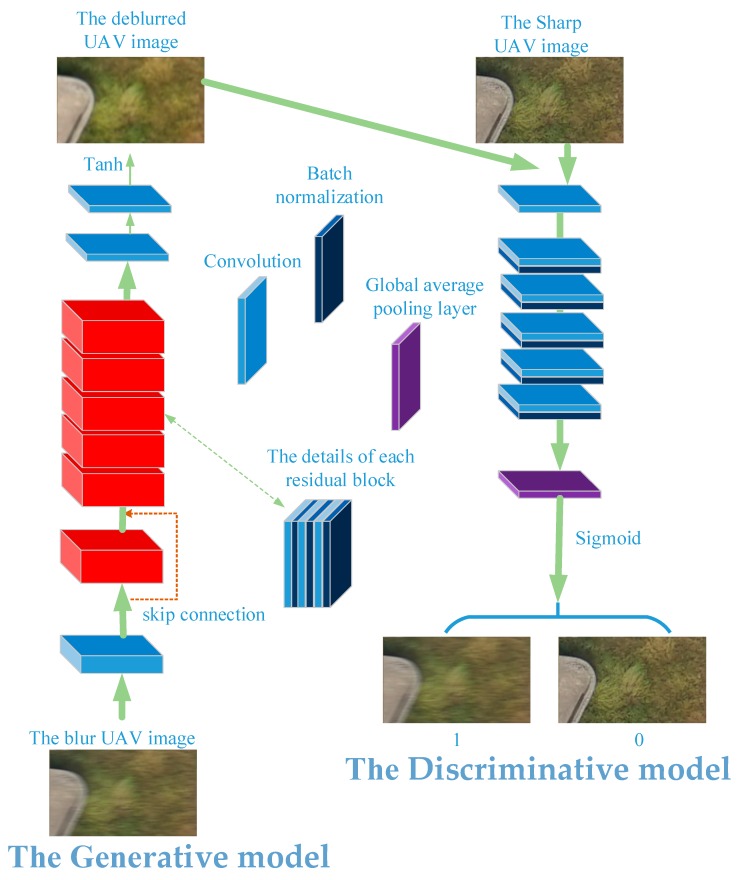
An overview of the proposed discriminative classifier. The networks include generative model G and discriminative model D.

The GAN-based networks take the image as input and output a percentage, indicating the probability that the input image is a blurred UAV image. In order to make the proposed networks provide better prior images, different blur levels and sizes of images are input into the networks. The proposed networks consist of generative model G and discriminative model D as shown in Figure 1.

We construct the generative model G containing six residual blocks, each of which consists of two convolutional layers and two batch normalization layers [55], and then a skip connection [56] is established. The residual block [57] can solve gradient vanishing at the structure of the deeper networks. Batch normalization makes distribution of input image back to the standard normal distribution, which makes the training faster and easier. The specific settings of each layer of the generative model D are as follows:

C(r,64)→C(64)B(r)C(64)B(r)SC→…4…→C(64)B(r)C(64)B(r)SC→C(64)→C(t,3)

where, C(r,64) denotes a set of convolutional layers with 64 feature maps and activation function, Relu; C(64)B(r)C(64)B(r)SC represents a residual block and B(r) is a batch normalizational layer with activation function, Relu, SC denotes a skip connection with a total of six residual blocks, while C(t,3) represents a convolutional layer with three feature maps and activation function, Tanh. The proposed discriminative model D is shown at the bottom part of Figure 1. The specific settings of each layer of the discriminative model are as follows:

C(lr,64)→C(128)BN(lr)→C(256)BN(lr)→C(512)BN(lr)→C(1024)BN(lr)→C(2048k)BN(lr)→SC→GA→SM

where, lr denotes the activation function, Leakyrelu; C(128)BN (lr) denotes a set of convolutional layers with 128 feature maps followed by batch-normalization with activation function, Leakyrelu, and GA is the global average pooling layer which converts feature maps into a percentage. SM indicates the sigmoid non-linear function. The feature maps are increased from 256 to 2048 which is suitable number for the pretrained VGG networks [58]. 

#### 3.1.2. The Loss Function

The primary aim of proposed method is to obtain effective classification networks, so when facing min-max adversarial game theoretic problem, we can fix generative model G and optimize discriminative model D. We optimize the proposed networks via Equation (2), as shown in:

(4) $$S(\rho )=\log(1-z_{i})(1-z_{i}^{t})-\frac{1}{M}\sum_{i=1}^{M}z_{i}^{t}\log(z_{i}^{})$$

where, *M* indicates input image, ρ indicates the optimized parameters by the proposed networks; $z_{i}^{t}=p(y;\rho )$, it is the output of the classifier that indicates the probability of the input image to be blurred, we let $z_{}^{t}$ = 0 for sharp images and $z_{}^{t}$ = 1 for blurred UAV images. 

### 3.2. Deblurring the UAV Images

After adding GAN-based image prior, the objective function Equation (2) of the deblurring UAV images converges and converted into Equation (5):

(5) $$\underset{L,f}{\min}∥L⊗f-B{∥}_{2}^{2}+\gamma ∥f{∥}_{2}^{2}+\mu ∥\nabla L{∥}_{0}^{}+\lambda d(L)$$

The deblurring process is modeled as an optimization problem by solving alternatively the latent image *L* and the blur kernel *f*, so we separated Equation (5) into the following Equations (6) and (7):

(6) $$\underset{L}{\min}∥L⊗f-B{∥}_{2}^{2}+\mu ∥\nabla L{∥}_{0}^{}+\lambda d(L)$$

(7) $$\underset{f}{\min}∥L⊗f-B{∥}_{2}^{2}+\gamma ∥f{∥}_{2}^{2}$$

#### 3.2.1. Estimating the Latent Image

Informed by existing methods [40,59], during the optimization of Equation (6), we use the half-quadratic splitting *L*_0_ minimization method and introduce auxiliary variables *j*, *k* that indicate image and image gradients respectively. Thus, the objective function can be rewritten as Equation (8):

(8) $$\underset{L,j,k}{\min}∥L⊗f-B{∥}_{2}^{2}+\theta ∥\nabla L-k{∥}_{2}^{2}+\omega ∥\nabla L-j{∥}_{2}^{2}+\mu ∥k{∥}_{0}^{}+\lambda d(j)$$

where, θ and ω are the penalty parameters. We can solve Equation (8) by alternatively minimizing *L*, *j*, and *k* while fixing other variables, thus avoiding the non-convex problem when directly minimizing $∥\nabla L{∥}_{0}^{}$ and d(L). 

The latent image *L* can be efficiently solved by fixing *g* and *u*. The solution for *L* is obtained by solving Equation (9) during each iteration:

(9) $$\underset{L}{\min}∥L⊗f-B{∥}_{2}^{2}+\theta ∥\nabla L-k{∥}_{2}^{2}+\omega ∥\nabla L-j{∥}_{2}^{2}$$

Equation (9) is a least squares minimization problem, whose solution is close to the solution for Equation (10):

(10) $$L=F^{-1}\left(\frac{\bar{F(f)}F(B)+\omega F(j)+\theta [(\bar{F(\nabla_{h})}F(k_{h})+\bar{F(\nabla_{v})}F(k_{v})]}{\bar{F(f)}F(f)+\omega +\theta (\bar{F(\nabla )}F(\nabla )}\right)$$

where, $F^{-1}(•)$ and F(•) indicate the Fast Fourier Transform (FFT) and inverse FFT, respectively; the $\bar{F(•)}$ is the complex conjugate operator; $k=(k_{h},k_{v})$, it is image gradients in horizonal and vertical directions, respectively; $\nabla_{h}$ and $\nabla_{v}$ indicate the horizontal and vertical differential operators, respectively.

Given the latent image *L*, we solve *j* and *k* separately with Equations (11) and (12):

(11) $$\underset{j}{\min}\omega ∥L-j{∥}_{2}^{2}+\lambda d(j)$$

(12) $$\underset{k}{\min}\theta ∥\nabla L-k{∥}_{2}^{2}+\mu ∥k{∥}_{0}^{}$$

In order to solve Equation (11), we use the back-propagation approach to compute the derivative of *d*(*j*) and update *j* by the gradient descent method:

(13) $$j^{\left(s+1\right)}=j^{s}+\varphi \left[\omega (j^{s}-L)+\lambda \frac{df(j^{s})}{d(j^{s})}\right]$$

where, s denotes the *s-*th iteration, s+1 denotes the *s* + 1-th iteration, φ denotes step width, $\frac{df(j^{s})}{d(j^{s})}$ denotes the differential of $j^{s}$.

Equation (12) is a pixel-wise minimization problem, thus, we solve (12) based on [60]. As shown in Equation (14):

(14) $$k=\left\{ \begin{array}{l} \nabla L,{\left|\nabla L\right|}^{2}\geq \frac{\lambda }{\theta }\\ 0,otherwise \end{array}\right.$$

#### 3.2.2. Estimating Blur Kernel

As in the existing methods [29,60], the kernel estimation methods based on gradients have been shown to be more accurate when given *L*. Therefore, we estimate the blur kernel using image gradients generated by Equation (15):

(15) $$\underset{f}{\min}∥\nabla L⊗f-\nabla B{∥}_{2}^{2}+\gamma ∥f{∥}_{2}^{2}$$

The solution of *j* is obtained by image pyramid which is similarly FFT [61]. After obtaining *f*, the negative elements were set to 0, and we normalize *f* so that the sum of its elements is 1. We alternatively solve (6) and (15) in iterations at each pyramid level. Algorithm 1 lists the pyramid level for coarse-to-fine solution.

**Algorithm 1:** Blur kernel estimation Input: Blur image Output: Blur kernel *f*
Initialize *f* with results from the coarser level. While iteration *i* ≤ 5 do Solve for *f* using Equation (14) end while

## 4. Experiments

### 4.1. Training Details of Discriminative Networks

In order to obtain latent images prior of UAV images deblurring, we constructed a training dataset of UAV images to learn the GAN-based classification networks. The training dataset of UAV images included two parts, one part was obtained by a CW-30 in Yangjiang City (Guangdong Province, China). The camera was a SWDC with a focal length of 50 mm, the size of each entire image was 8206 × 6078 pixels and the flying height was 600–800 m; the other part of the training dataset was obtained by a CW-30 in Guiyang City (Guizhou Province, China). The camera was a H5D-50 with a focal length of 50 mm, the size of each entire image is 8176 × 6132 pixels and the flying height was 600–800 m. For the convenience during training, we cut entire images into smaller images by using Photoshop. In order to obtain blurred UAV images, we added realistic synthesized blur at different sizes and intensities to 1000 sharp UAV images at 320 × 320 pixels. The discriminative networks were trained on an Nvidia GRID M60-8Q (8G) GPU using the tensorflow framework and the number of training iterations was 80 k. Due to the limited memory of the computer, the batch size of our experiment was 1. We used Aadm as an optimization algorithm and set the initial learning rate to 0.002 which was decreased by a factor of 10 for every 100 epochs. 

During training, all strides were set to 1 in the generative model G and all other convolution kernel sizes were 3 × 3 except for the fact that the last convolution kernel sized at 1 × 1. In the discriminative model D, the first six layers were composed of kernels of size 4 × 4 with a stride 2; the next layer was composed of kernels of sized 1 × 1 with a stride 1 and the last two layers were composed of kernels of size 3 × 3 with a stride 1. In generative model G and discriminative model D, all padding modes were padded edges of kernels. 

### 4.2. Comparison and Qualitative Evaluation

**Figure 2:**
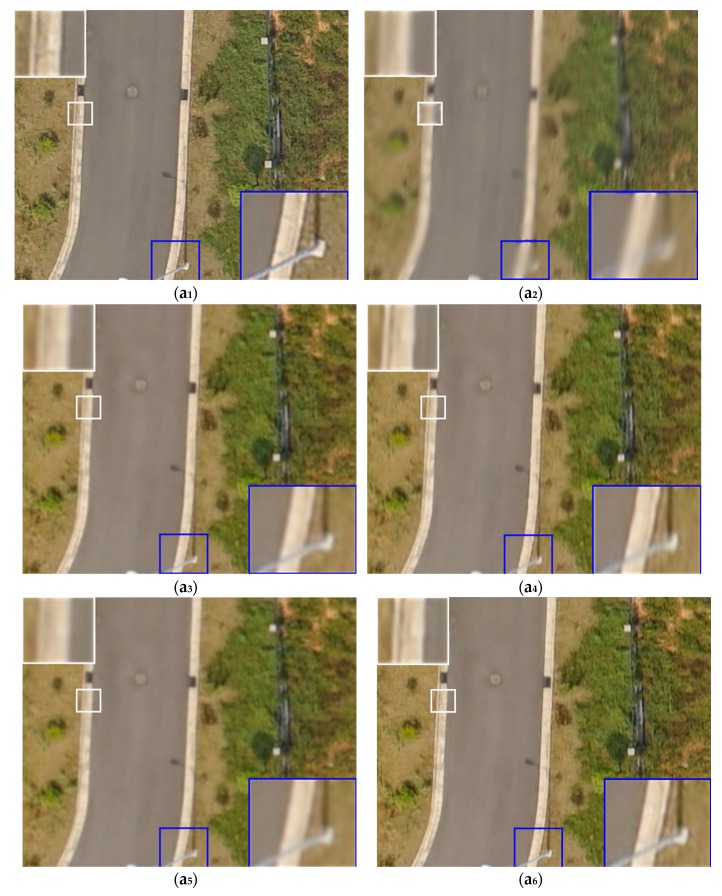

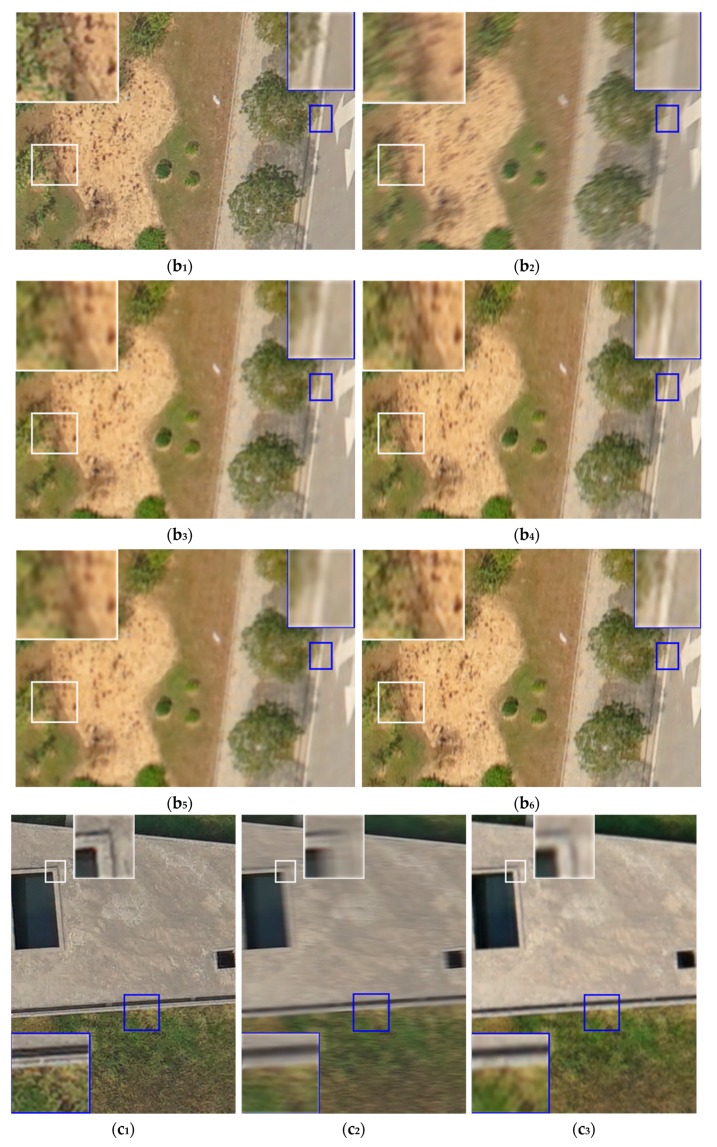

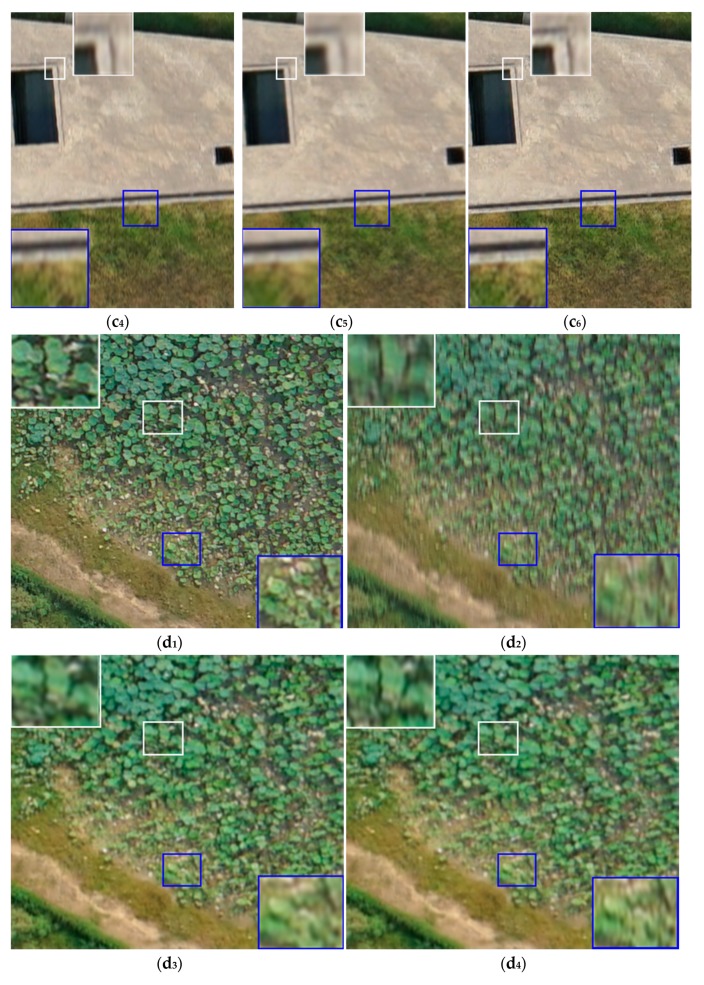

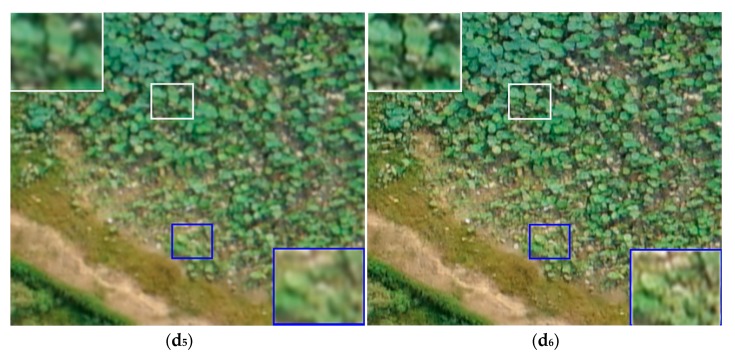
Test results from the four deblurring methods for UAV images (the proportional scale of figure is 49%), incluing several ground objects. In each group of images, the 1st images (**a_1_**–**d_1_**) are the ground truth; the 2nd images (**a_2_**–**d_2_**) are synthetically blurred image; and the 3rd images (**a_3_**–**d_3_**) present the deblurring results from method [17]. The 4th images (**a_4_**–**d_4_**) present the deblurring results from method [59]; the 5th image (**a_5_**–**d_5_**) present the deblurring results from method [38]; and the 6th images (**a_6_**–**d_6_**) present the deblurring results from the proposed method.

After the training process of the GAN-based networks converge, the trained model was used as the latent prior image. In all the experiments, we set μ = 0.005, λ = 0.005, and γ = 2. We evaluated the proposed algorithm on synthetic blurred UAV images and real blurred UAV images. The testing dataset was photographed in other mapping areas, which is obtained by a CW-10 in Wuhan City (Hubei Province, China) where the camera was an ILCE-7R with a focal length of 28 mm, the size of entire image was 7360 × 4916 pixels and the flying height was 400–600 m. One part of the testing dataset included originally sharp UAV images. We artificially added synthetic blurring with different sizes and intensities to these data, as shown in Figure 2. The other part of the testing dataset were the real blurred UAV images. The proposed method was compared qualitatively and quantitatively to method [17], method [59] and method [38]. We chose the method [17] because it is related to remote sensing images deblurring, and therefore comparable. The method [59] and method [38] were seen as a widespread reference, and they closely related to our work, so they can be used as a comparatively valuable references for this paper.

#### 4.2.1. Comparison of the Synthetic Blurred UAV Image Results

In Figure 2, we provide the experimental results for four UAV images of different ground objects to which synthetic blurring of different sizes and intensities were added. The labels a_1_–d_6_ indicate the real clean UAV images, the labels a_2_–d_2_ indicate the synthetically blurred UAV image (with synthetic blur of different sizes and intensities added), the labels a_3_–d_3_ indicate the deblurred UAV images from method [17]. The labels a_4_–d_4_ indicate the deblurred UAV images from method [59], the labels a_5_–d_5_ indicate the deblurred UAV images using method [38], and the labels a_6_–d_6_ indicate the deblurred UAV images from proposed method. In each of the images, small rectangles in white and blue identify areas enlarged for examination shown in the larger white and blue rectangles inserted each series of images. 

**Figure 3:**
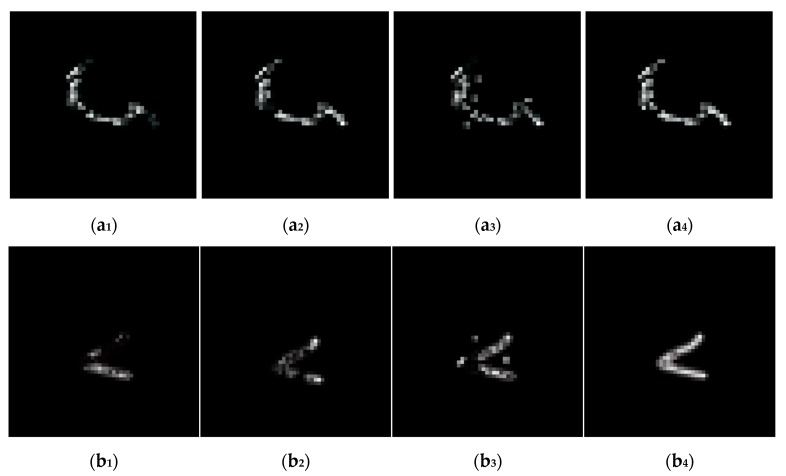

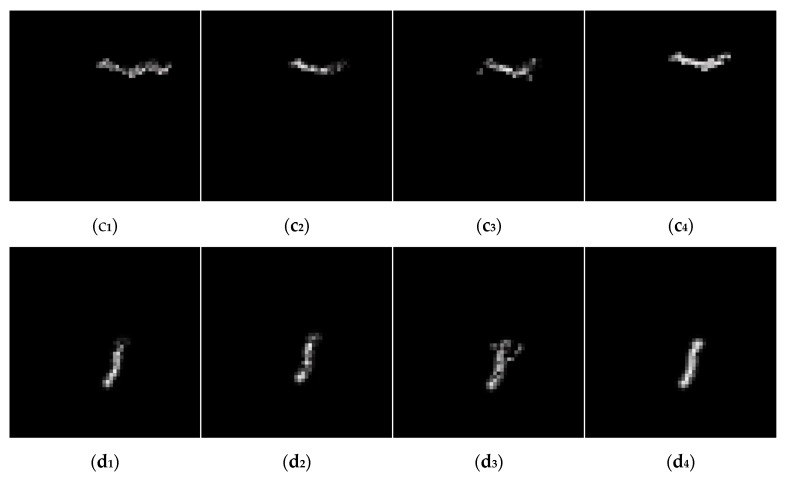
The estimated blur kernels of deblurring UAV images shown in Figure 2. The images (**a_1_**–**a_4_**) are the estimated blur kernels of (**a_3_**–**a_6_**) seen in Figure 2, the images (**b_1_**–**b_4_**) are the estimated blur kernels of (**b_3_**–**b_6_**) found in Figure 2, the images (**c_1_**–**c_4_**) are the estimated blur kernels of (**c_3_**–**c_6_**) seen in Figure 2, and the images (**d_1_**–**d_4_**) are the estimated blur kernels of (**d_3_**–**d_6_**) found in Figure 2.

Figure 3 shows the estimated blur kernels of deblurring UAV images in Figure 2, which allow visually comparison of blur kernel estimation. It seems obvious that the blur kernels obtained by proposed method have more continuous blobs. The blur kernels obtained by proposed method are much more plausible in visually comparison.

A qualitative visual comparison of images in Figure 2, produced by the proposed method and the other tested methods shows that restores more distinct ground edges and more detailed ground objects textures. Meanwhile, the deblurred UAV images obtained by proposed method are closer to the true ground objects.

In order to quantitatively evaluate the proposed method, we employ Structural Similarity Index (SSIM) [62] and Image Quality measure (FM) [63] for comparative evaluation. FM is image sharpness measure for blurred images in frequency domain, it been calculated by the Equation (16). The specific details of the algorithm about FM can be seen in [63]:

(16) $$\mathrm{Image}\;\mathrm{Quality}\;\mathrm{Measure}\;\left(\mathrm{FM}\right)=\frac{T_{H}}{M\times N}$$

Table 1 lists the SSIM values of deblurred UAV images obtained by several deblurring methods, respectively. In Table 1, row 1 denotes the tested methods, column 1 denotes four UAV images of different ground objects, column 2 denotes the SSIM values of deblurred images obtained by method [17], column 3 denotes the SSIM values of deblurred images obtained by method [59]. Column 4 denotes the SSIM values of deblurred images obtained by method [38], and column 5 denotes the SSIM values of deblurred images obtained by proposed method. Table 2 is similar to Table 1, but shows results using FM for the tested methods.

It can be seen in Table 1 and Table 2, the proposed method has the highest SSIM and FM, this is consistent with the visual effects of the deblurred UAV images.

#### 4.2.2. Comparison of the Real Blurred UAV Image

**Figure 4:**
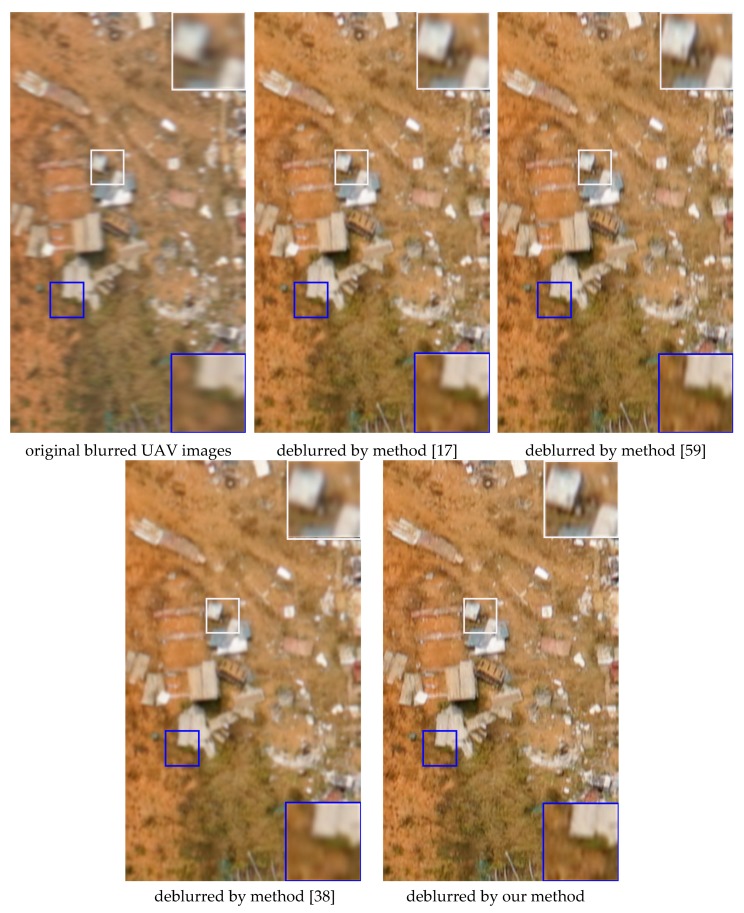

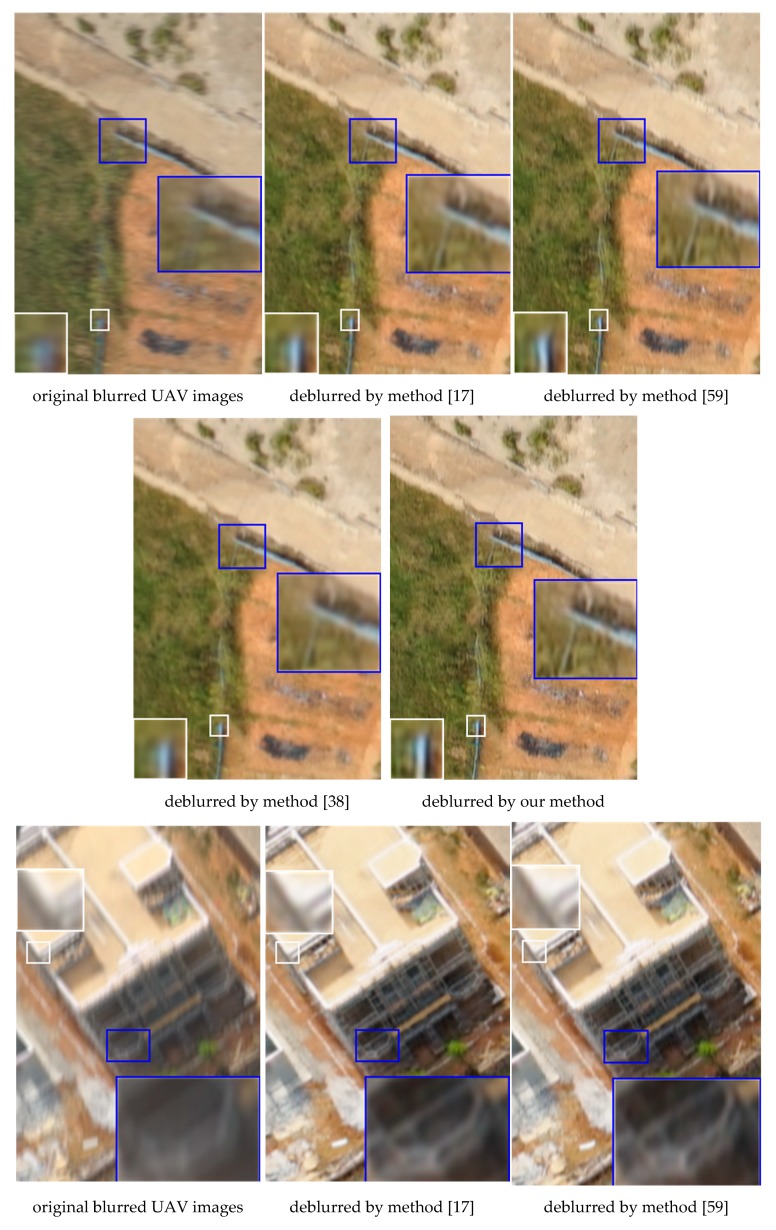

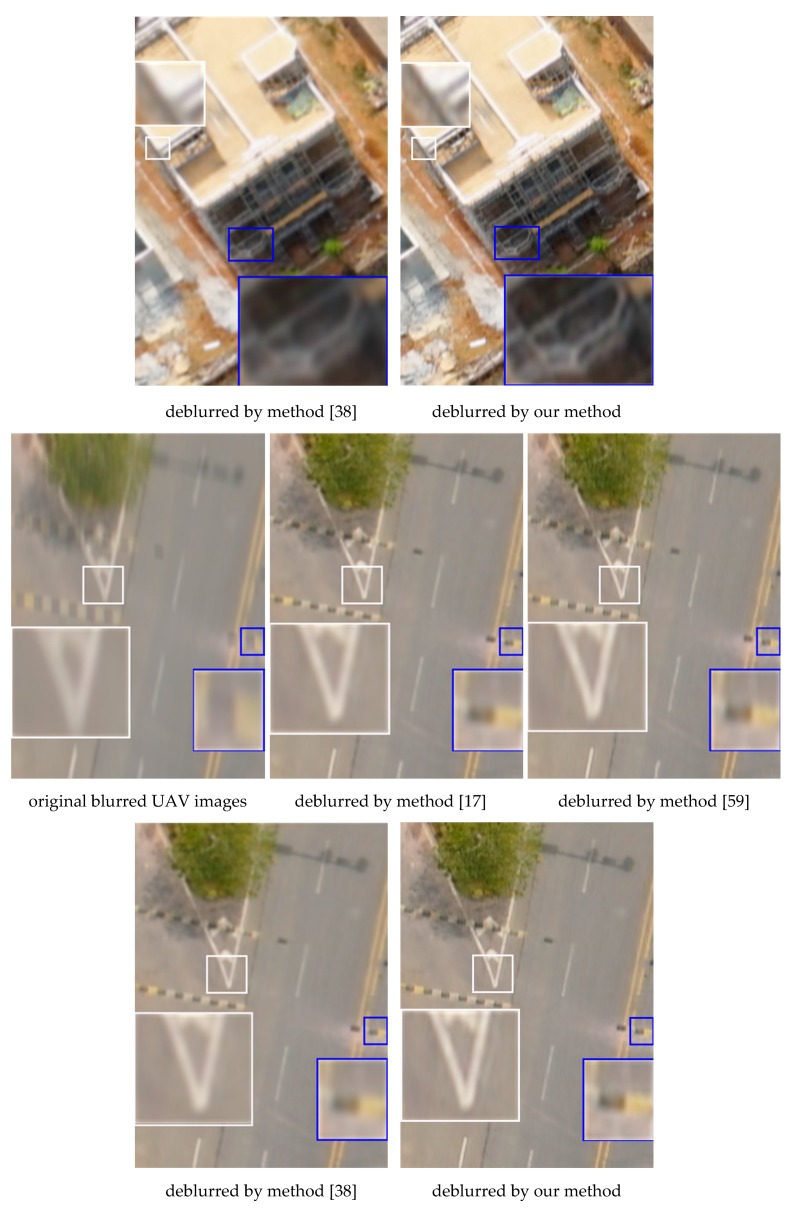

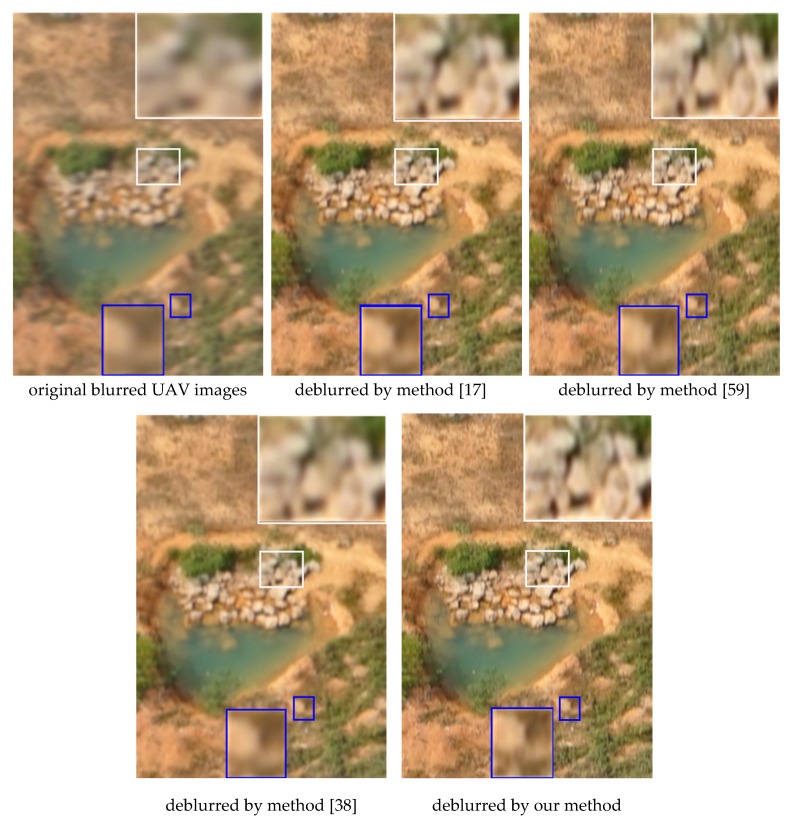
Testing results of several deblurring methods for real blurred UAV images (the proportional scale of figure is 49%). In each group of images, the 1st images are the original blurred UAV images; the 2nd images present the deblurring results from method [17]; and the 3rd images present the deblurring results from method [59]. The 4th images present the deblurring results from method [38]; the 5th images present the deblurring results from the proposed method.

We evaluated the proposed method on the real blurred UAV images, consisting of five UAV images with different ground objects. Figure 4 shows the deblurred results for several real UAV images processed with the proposed algorithm and other test methods. 

**Figure 5:**
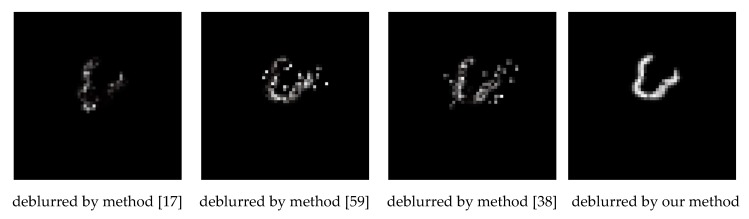

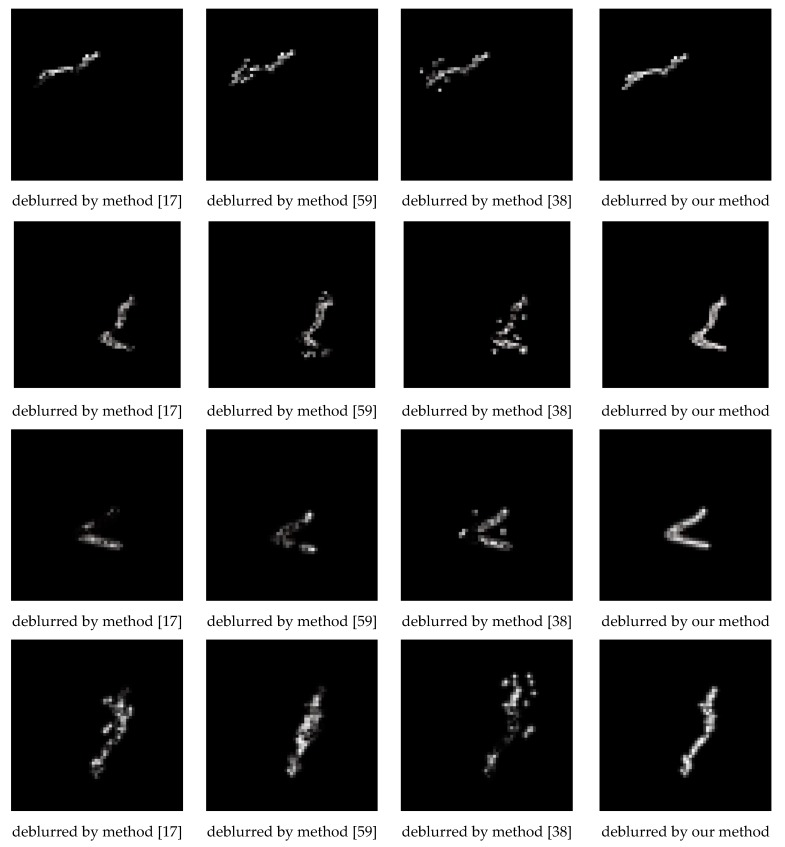
The estimated blur kernels for deblurred UAV images in Figure 4. The images of column 1 are the estimated blur kernels of deblurred UAV images by method [17], The images of column 2 are the estimated blur kernels of deblurred UAV images by method [59], The images of column 3 are the estimated blur kernels of deblurred UAV images by method [38], and the images of column 4 are the estimated blur kernels of deblurred UAV images by our method.

It can be seen that the deblurred image output by the proposed algorithm contain clearer ground objects textures and generates fewer artifacts. Figure 5 shows the estimated blur kernels of deblurring UAV images in Figure 4, which shows the blur kernels estimation for visual inspection.

Table 3 presents the FM values of deblurred UAV images obtained by several deblurring methods, respectively. In Table 3, row 1 denotes the tested methods, column 1 denotes 5 real UAV images of different ground objects, column 2 denotes the FM values of deblurred images obtained by method [17], column 3 denotes the FM values of deblurred images obtained by method [59]. Column 4 denotes the FM values of deblurred images obtained by method [38], and column 5 denotes the FM values of deblurred images obtained by proposed method. It can be observed in Table 3, the proposed method has the highest FM, this is consistent with the visual effects of the real deblurred UAV images.

The better deblurred images tend to restore better the local features found in the UAV images. If conducting a matching method based on local features to match a deblurred image with a sharp image in the same region, the better matching results should be able to demonstrate the better deblurring results, so we try to compare the deblurring results from the perspective of image matching. Because the UAV platform for acquiring images consists of multiple cameras—the left-view, the front-view, the right-view, the back-view, the down-view camera—this allows us to obtain images from different angles in the same mapping area. In order to further verify our method, the deblurred UAV images gathered by several methods are matched with the clear images of different cameras in the same mapping areas so that the results of the matching experiments are employed to compare the results of deblurring.

SIFT [64] is a well-known matching algorithm in image matching, which is based on matching methods of image local features. The quality of the matching results can often reflect the similarities of the local characteristics of the two images, and the number of matching pairs is the standard for judging the quality of matching results. Therefore, in this article, we compare the several deblurring methods using the number of matching point pairs based on SIFT.

**Figure 6:**
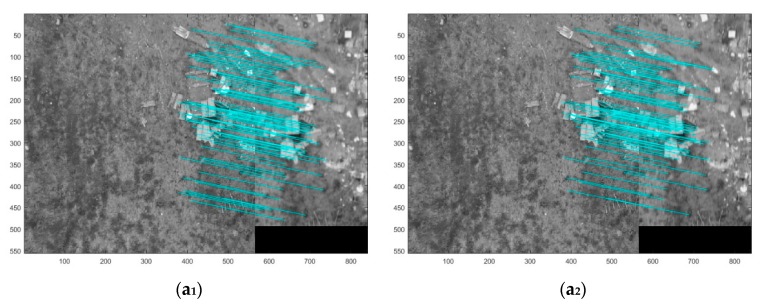

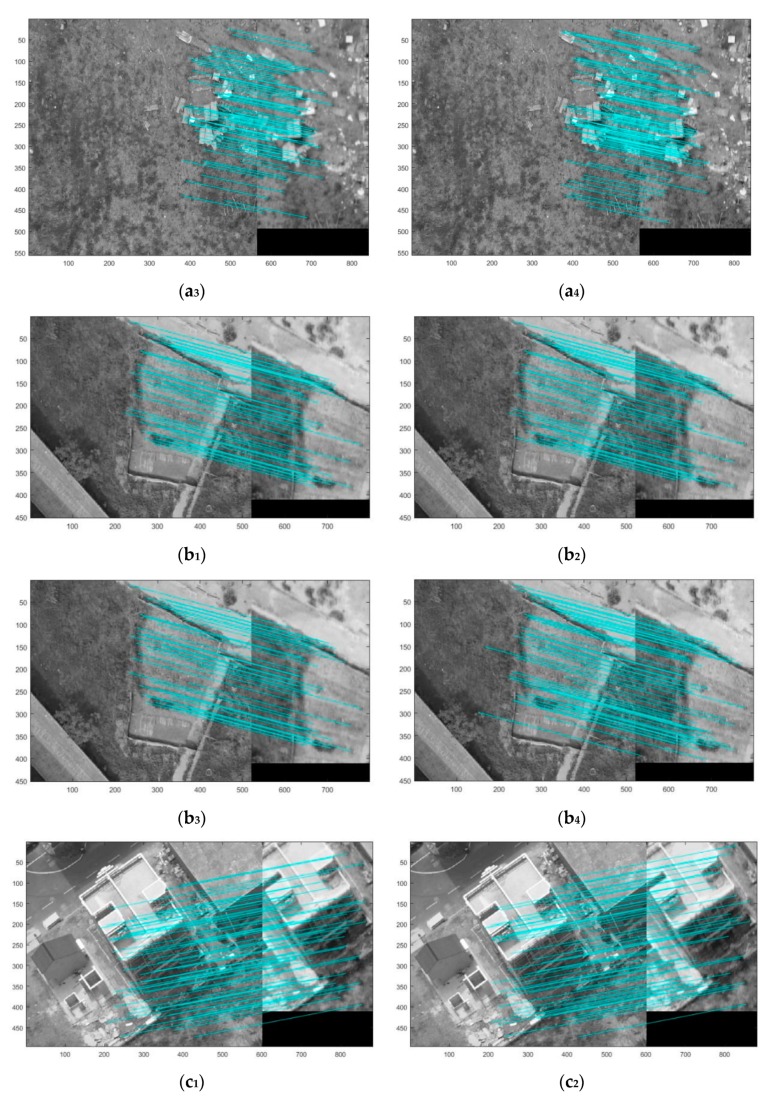

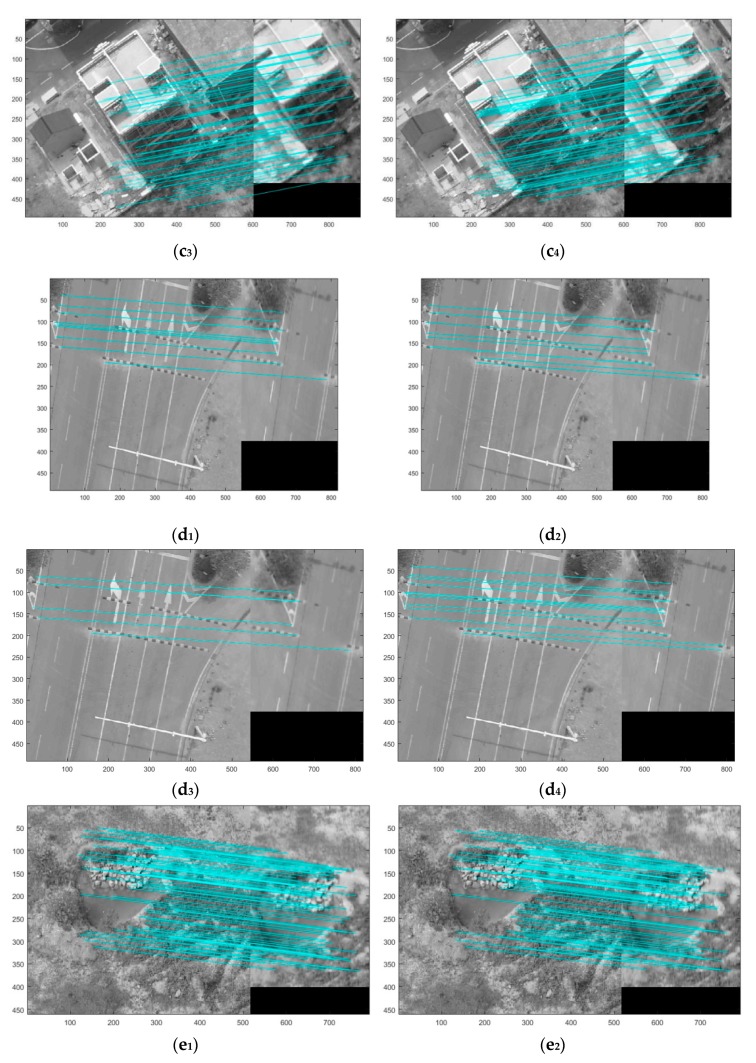

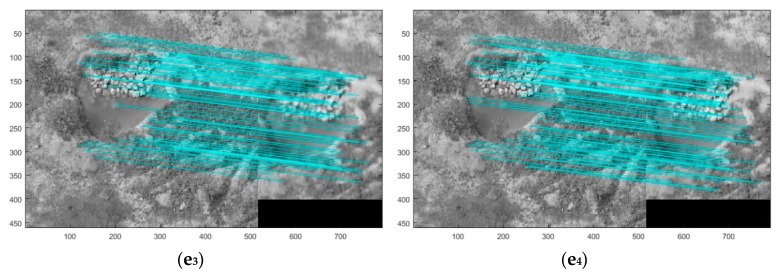
In each matching image (the proportional scale of figure is 30%), the left image is the sharp UAV image, and the right image is the deblurred UAV image. The 1st images (**a_1_**–**e_1_**) present matching results from SIFT and the deblurred UAV images using method [17] and sharp UAV images. The 2nd images (**a_2_**–**e_2_**) present matching results from SIFT and the deblurred UAV images using method [59] and the sharp UAV images. The 3rd images (**a_3_**–**e_3_**) present matching results from SIFT and the deblurred UAV images created by method [38] and sharp UAV images; and the 4th images (**a_4_**–**e_4_**) present matching results from SIFT and the deblurred UAV images using proposed method and the sharp UAV images.

Figure 6 shows the matching results of a sharp UAV image with different camera views and deblurred UAV images of the same region by the four tested methods. Table 4 shows the number of correct matching pairs about Figure 6. It can be observed that the deblurred UAV images generated by proposed method obtain more correct matching pairs than other methods in number.

Through comparative experiments with the real blurred UAV images and synthetically blurred UAV images, it can be seen that the deblurred images obtained by the proposed method can obtain better deblurring results. In the comparative experiments with synthetic data, the proposed method obtained the highest SSIM and FM values. In the comparative experiments with real data, our method can better restore the local features of real blurred UAV images and obtained clearer ground objects textures.

## 5. Conclusions

In this paper, we utilized a trained discriminative model as a classifier which can effectively distinguish between blurred and clear UAV images in UAV image deblurring. The trained classifier is used as prior image information to continuously optimize the blind deblurring of UAV images to get better deblurring results. In comparative experiments of simulated and real blurred UAV images, the proposed method has obtained better results in various ground objects. In the process of training the discriminative model, we input UAV images with different intensities of blurring, which make the data-driven prior image information more applicable when deblurring real UAV images.

## Figures and Tables

**Table 1:** Quantitative measurement results using SSIM on synthetic blurred UAV testing images.

Images	Method [17]	Method [59]	Method [38]	Ours
a	0.8776	0.8752	0.8548	0.8824
b	0.8749	0.8709	0.8492	0.8782
c	0.8185	0.8157	0.7832	0.8232
d	0.8298	0.8285	0.7853	0.8342
Average results ^1^	0.8518	0.8496	0.8181	0.8577

^1^ The average results of 50 synthetic blurred UAV test images.

**Table 2:** Quantitative measurement results using FM on synthetic blurred UAV testing images.

Images	Method [17]	Method [59]	Method [38]	Ours
a	0.0581	0.0522	0.0497	0.0624
b	0.0479	0.0385	0.0413	0.0545
c	0.0665	0.0541	0.0592	0.0712
d	0.0526	0.0495	0.0436	0.0597

**Table 3:** Quantitative measurement results using FM on real blurred UAV testing images.

Images	Method [17]	Method [59]	Method [38]	Ours
a	0.0322	0.0276	0.0241	0.0392
b	0.0257	0.0188	0.0239	0.0336
c	0.0349	0.0283	0.0217	0.0424
d	0.0276	0.0214	0.0158	0.0297
e	0.0495	0.0473	0.0394	0.0561

**Table 4:** The comparison of the correct matching pairs which are between deblurred images and the corresponding sharp UAV images is obtained by SIFT.

Images	Method [17]	Method [59]	Method [38]	Ours
a	99	91	84	115
b	44	45	40	57
c	72	67	64	81
d	13	11	7	16
e	88	81	77	93
